# Supplementary material for: Signal peptidase complex mediates rotavirus VP7 processing and virion assembly
Source: bioRxiv. 2025 Nov 4:2025.11.04.686468. Preprint. [Version 1] doi: 10.1101/2025.11.04.686468 (PMC12637713; doi:10.1101/2025.11.04.686468)
Supplement: 1 [file NIHPP2025.11.04.686468v1-supplement-1.pdf]

bioRxiv preprint doi: https://doi.org/10.1101/2025.11.04.686468; this version posted November 4, 2025. The copyright holder for this preprint (which was not certified by peer review) is the author/funder, who has granted bioRxiv a license to display the preprint in perpetuity. It is made available under aCC-BY 4.0 International license.

S1 Fig. Localization of host protein and viral protein *in vitro*.

Plasmids expressing -VP7 and REEP5, SEC11C, SPCS2, -VP3 and PFDN4, -NSP1 and SAMD9, -VP6 and ECE1 were co-transfected into HEK293 cells, respectively, and subjected to IFA detection. Viral proteins were tagged with GFP, shown as green fluorescence, while host proteins were tagged with RFP, shown as red fluorescence. Nuclei were counterstained with DAPI. Scale bar, 170 μm.

S2 Fig. Knockdown efficiency of SPC components in WT and *SPCS1* KO HEK293T cells.

WT and *SPCS1* KO HEK293T cells were transfected with siRNAs targeting against *SPCS2*, *SEC11A*, *SEC11C* and a scrambled siRNA at the concentration of 20 nM. At 72 hours post-transfection, cells were collected for RNA extraction, and relative mRNA expression levels were measured by qPCR. The relative expression was normalized to GAPDH. Results are the average of data from two independent experiments and plotted

bioRxiv preprint doi: https://doi.org/10.1101/2025.11.04.686468; this version posted November 4, 2025. The copyright holder for this preprint (which was not certified by peer review) is the author/funder, who has granted bioRxiv a license to display the preprint in perpetuity. It is made available under aCC-BY 4.0 International license.

as mean ± SD. Statistical significance was determined by two-way ANOVA with Sidak's multiple comparisons test (*, $P < 0.05$, **, $P < 0.01$; ***, $P < 0.001$, ****, $P < 0.0001$).

S3 Fig. RNA and protein level in wildtype and *SPCS1* KO Huh7.5 cells during rotavirus infection.

(A) WT and *SPCS1* KO Huh7.5 cells were infected with RRV at an MOI of 3. At 4, 8, 12 hpi, the cells were collected for qRT-PCR analysis of viral mRNA level by detecting NSP5. NSP5 level was normalized to GAPDH. The result was representative of one independent experiment. (B) WT and *SPCS1* KO Huh7.5 cells were infected with RRV at an MOI of 3. At 4, 8, 12 hpi, the infected and mock cells were harvested for western blot analysis of viral protein levels by detecting VP6.

S4 Fig. Transmission electron microscopy images of viral particle morphology.

Transmission electron micrographs of RRV particles in HEK293T cells. (A) Single-layered particles indicated by blue arrows surrounding the viroplasm. 'V' indicates the viroplasm. (B) DLP, indicated by blue arrow. (C) TLPs, indicated by blue arrow. (D) The budding process of DLP morphing into TLP, indicated by blue arrow, 'V' indicates the viroplasm. Scale bar, 100 nm.

bioRxiv preprint doi: https://doi.org/10.1101/2025.11.04.686468; this version posted November 4, 2025. The copyright holder for this preprint (which was not certified by peer review) is the author/funder, who has granted bioRxiv a license to display the preprint in perpetuity. It is made available under aCC-BY 4.0 International license.

Dataset 1. Raw data of LC-MS/MS for DS-1 VP7

Peptide-spectrum matches were identified against the NCBI human protein database, the raw data were listed in three columns. The Spectra table lists individual peptides with their observed and theoretical precursor masses, charge states, modifications. The Proteins table summarizes all identified proteins, including cumulative log-probabilities, best scores, total spectral counts, sequence coverage, and numbers of unique and modified peptides. The Summary sheet includes information on the data file, search parameters. These raw data collectively describe the peptide and protein composition detected in the analyzed sample.
